# Anti-*Staphylococcus aureus* activity of methanol extracts of 12 plants used in Cameroonian folk medicine

**DOI:** 10.1186/s13104-015-1663-1

**Published:** 2015-11-24

**Authors:** Leonard Sama Fonkeng, Raymond Simplice Mouokeu, Christopher Tume, Guy Sedar Singor Njateng, Monique Odette Kamcthueng, Nfozon Jinette Ndonkou, Jules-Roger Kuiate

**Affiliations:** Laboratory of Microbiology and Antimicrobial Substances, Faculty of Science, P.O. Box 67, Dschang, Cameroon; Institute of Fisheries and Aquatic Sciences, University of Douala, P.O. Box 7236, Douala, Cameroon

**Keywords:** Medicinal plant, *Staphylococcus aureus*, Antibacterial activity, Cameroonian folk medicine

## Abstract

**Background:**

The emergence of bacterial infections including those associated with *Staphylococcus aureus* causes a benefit of interest to medicinal plants as an effective means of control. The present study was designed to investigate the activities of 12 selected Cameroonian medicinal plants against *S. aureus* isolates.

**Methods:**

The plant extracts were prepared by maceration in methanol at laboratory temperature. Qualitative phytochemical analysis was performed by chemical reaction methods. The broth microdilution method was used to evaluate the activities of plant extracts against 11 *S. aureus* clinical isolates.

**Results:**

*Dacryodes edulis* was found to have significant antibacterial activity on all the *S. aureus* isolates (MIC = 64–256 µg/ml). *Occimum gratissimum* revealed significant inhibitory activity on 9 of the 11 isolates while *Commelina erecta* and *Spilanthes filicaulis* revealed similar results on 6 of the 11 clinical isolates.

**Conclusion:**

The present findings showed that *D. eduli, O. gratissimum,C. erecta* and *S. filicaulis* possess interesting inhibitory properties against *S. aureus* species. These plants could therefore be good candidates to overcome infectious diseases associated with these microorganisms.

## Background

Infectious diseases are becoming a major cause of human and animal mortality and morbidity. This is further aggravated by the rapid development of multi-drug resistance, limited antibacterial spectrum and adverse effects of available antimicrobial agents [1]. Human pathogenic bacteria include amongst others *Staphylococcus aureus*; a major cause of bacteremia, associated with higher morbidity and mortality compared to other bacteremia-causing pathogens [2]. The burden of *S. aureus* bacteremia, particularly methicillin-resistant *S. aureus* bacteremia is due to the fact that, it is highly expensive in terms of cost and resource. The incidence of *S. aureus* bacteremia and its complications has increased abruptly in recent years because of the increased frequency of invasive procedures which has led to great number of immunocompromised patients and resistance of *S. aureus* strains to available antibiotics. This changing epidemiology of *S. aureus* bacteremia, in combination with the inherent virulence of the pathogen, is commanding an urgent need for improved strategies and better antibiotics to prevent and treat *S. aureus* bacteremia [3, 4].

The investigation of certain indigenous plants for their antimicrobial properties may yield useful results. This has consequently increased the attention and demand given to antimicrobials derived from the plants [5]. Natural products, either as pure compounds or as standardized plant extracts, provide exceptional opportunities for new drug leads because of the unmatched chemical diversity of naturally derived compounds [6, 7]. The medicinal value of plants is associated with some chemical substances also known as phytochemicals that produce a definite physiological action on humans. The present study was designed to investigate the activities of 12 selected Cameroonian medicinal plants against *S. aureus* clinical isolates.

## Methods

### Collection and identification of plant samples

Twelve plant samples were used in this study. They were collected either from Santchou or Dschang in April 2011, Menoua division, West Region of Cameroon. The taxonomical authentication of identity was undertaken by a botanist at the National Herbarium of Cameroon in Yaounde where voucher specimens were deposited. For each plant, the part used, the identification code, previous studies and ethnobotanical surveys are presented in Table 1.

**Table 1:** Information on the plants used, yields of extraction and report on evidence of their activities

Plant name	Parts use tradionally	Locality of harvest	Yields (%)	Ethnobotanical relevance	Identification code	Previous scientific studies
*Acalypha frutucosa* Forssk	Leaf	Santchou	3.43	Skin infections and diarrhoea	33,034/HNC	Antimicrobial and antioxidant activity [8]
*Aspilia africana* CD/Adams	Leaf	Santchou	2.51	Wound treament	16,935/SRF Cam	Antiulcer activity [9]
*Commelina Erecta*	Leaf	Santchou	1.56	Eczema and skin infection treatment	22,595/SRF Cam	/
*Dacryodes edulis* (Don) H.J Lam	Leaf	Santchou	4.44	Stomach ache	17,234/SRF Cam	Phytochemical studies and antimicrobial activity [10]
*Drymaria cordata* Willd		Dschang	1.02	Headache	20,550/SRF Cam	Cytotoxic activity [11]
*Eremomastax speciosa* Cufod	Leaf	Dschang	2.26	Nappy rash treatment	36,228/HNC	Antidiarrhoea and antimicrobial activity [12, 13]
*Kalanchoe crenata* Andr	Leaf	Santchou	10.92	Ear infection, rheumatism and inflammatory treatment	50,103/YA Cam	anti-inflamatory and antibacterial activity [14, 15]
*Occimum gratissimum* linn Hochst	Leaf	Santchou	9.54	Food plant	42,850/HNC	Antimicrobial and phytochemical studies [16, 17]
*Portulaca oleracea*	Leaf	Dschang	4.61	Food plant	17,542/SRF Cam	Antimicrobial and phytochemical studies [18]
*Scoporia dulcis* Linn	Leaf	Santchou	8.12	Stomach ache	22,595/SRF Cam	Antidiabetic activity [19]
*Sida veronicifolia* Linn	Leaf and steam	Santchou	1.11	Stomach ache	20,859/SRF Cam	Antioxydant activity [20]
*Spilanthes filicaulis* C .D. Adams	Leaf and steam	Santchou	5.54	Headache, fontanel, teeth pain, angina	20,447/SFR Cam	Phytochemical studies and antiulcer activity [9]

### Preparation of plant extracts and preliminary qualitative phytochemical screening

The leaves or the stems of each plant were air-dried at room temperature (20 ± 4 °C) before grinding to powder with a mechanical grinder. The powder (150 g) was macerated in methanol (300 ml) for 4 days with a three times daily shaking, the mixture was then filtered using Whatmann filter paper No. 1. The filtrate was concentrated at 50 °C under reduced pressure using a rotary evaporator (Buchi R-200) and further dried using a vacuum concentrator (SC250EXP).

The qualitative phytochemical analysis was performed following standard methods [21].

### Microorganisms

A total of 11 clinical isolates of *S. aureus* were used. They were isolated locally on Mannitol salt agar slant (Conda, Madrid, Espagne) from patients with urogenital infections. The species was confirmed following morphological observations and biochemical tests [22]. These micro-organisms were maintained in agar slants.

### Antimicrobial susceptibility testing

Luria–Bertani agar (Himedia, India) was used for the upkeeping of the isolates, whereas Luria–Bertani broth (LBB) was used for antimicrobial susceptibility testing using broth microdilution method.

The antibacterial activity was investigated by determining the minimum inhibitory concentrations (MICs) and the minimum bactericidal concentrations (MBCs). The MICs value of the plant extracts were determined using a rapid p-Iodonitrotetrazolium (INT) chloride (Sigma-Aldrich, France) colorimetric assay [23]. Briefly, stock solutions of plant extracts were prepared in 5 % (v/v) dimethylsulfoxide (DMSO) solution (Fisher chemicals, France). The solution obtained was then diluted with LBB (Himedia, India) to give a final concentration of 4096 µg/ml. 100 ml of each extract solution was introduced into the first three wells of 96-wells microtitre plate containing 100 µl of LBB and further twofold serially diluted to obtain concentrations ranging from 1024 to 8 µg/ml. 100 ml of bacterial suspensions of about 1.5 × 10^8^ CFU/ml following Mc Farland turbidity standard no. 0.5, 100 times diluted, were introduced into each well containing 100 µl mixture of LBB and extract. The final concentration of DMSO was less than 1 %. Wells containing LBB, inoculum and DMSO at a final concentration of 1 % served as the negative control. Ciprofloxacin (Sigma-Aldrich, France) was used as reference antibiotic. The plates were covered with a sterile plate sealer and then agitated with a shaker. They were further incubated at 35 °C for 24 h. Upon incubation, 40 µl of 2 % INT solution were added in each well. Viable bacteria reduced the yellow dye of INT to pink. All the concentrations that did not show color change were considered, and the smallest was noted as MIC value of each extract on the isolate.

For the well that did not received INT, 50 µl of solution of the corresponding well that did not present color change was withdrawn out and seeded into the wells of new plates containing 150 µl of newly LBB prepared. The mixture was further incubated at 35 °C for 48 h. After the incubation period, 40 µl of INT solution were introduced in each well. The MBCs were considered as the lowest concentration of the extract that prevents INT color change [23].

## Results

### Qualitative phytochemical composition

Freshly prepared extracts were subjected to phytochemical screening for various constituents. The results revealed the presence of Phytochemical compounds including alkaloids, anthocyanins, anthraquinones, flavonoids, phenols, tannins and triterpenes (Table 2). Only *Dacryodes edulis* extract was found to contain saponins.

**Table 2:** Qualitative phytochemical composition of the plant extracts

	*A. africana*	*A. frutucosa*	*C. erecta*	*D. cordata*	*D. edulis*	*E. speciosa*	*K. crenata*	*O. gratissimun*	*P. oleracea*	*S. dulcis*	*S. filicaulis*	*S. veronicifolia*
Phenols	+	+	+	+	+	+	+	+	–	+	–	+
Saponins	–	–	–	–	+	–	–	–	–	–	–	–
Tannins	–	+	–	+	+	–	+	–	–	–	–	–
Flavonoids	–	–	+	+	+	+	+	+	–	+	+	+
Anthraquinones	–	+	–	–	+	–	–	–	+	–	–	–
Anthocyanins	–	–	–	+	+	+	–	+	+	+	+	+
Alcaloids	+	–	+	–	+	–	–	–	+	+	–	–
Sterols	+	–	+	–	+	–	+	+	+	+	–	+
Triterpenes	+	–	+	+	–	+	+	–	+	+	–	+

*+* present*; –* absent

### Antibacterial activity

The antibacterial activities of the 12 plant extracts on *S. aureus* isolates are presented in Table 3. *D. edulis* and *Occimum gratissimum* with MIC values ranging from 64–256 µg/ml were found to have the best inhibitory activity on almost all the tested microorganisms. *Scopariadulcis*, *Spilanthes filicaulis,Commelina erecta* and *E. spciosa* with MIC = 64–512 µg/ml were found to have similar antibacterial activity, being more active compared to *Kalanchoe crenata* (MIC = 512–1024 µg/ml). *Aspilia africa, Drymaria cordata*, *Portulaca oleracea* and *Sida veronicifolia* were almost inactive on these microorganisms.

**Table 3:** Minimal inhibitory concentrations and minimal bactericidal concentrations (µg/ml) of plant extracts against *Staphylococcus aureus* isolates

	Staph 23 JN	Staph 55 M	Staph 67 JN	Staph 18 JL	Staph 79 M	Staph 58 M	Staph 22 JN	Staph 70 M	Staph 02 JN	Staph 94 M	Staph 75 N
*A. africa*											
CMI	>1024	>1024	>1024	>1024	>1024	>1024	>1024	>1024	>1024	>1024	>1024
CMB	/	/	/	/	/	/	/	/	/	/	/
CMB/C MI	/	/	/	/	/	/	/	/	/	/	/
*A. frutucosa*											
CMI	>1024	512	>1024	>1024	>1024	>1024	>1024	>1024	>1024	512	>1024
CMB	/	1024	/	/	/	/	/	/	/	1024	/
CMB/CMI	/	2	/	/	/	/	/	/	/	2	/
*C. erecta*											
CMI	>1024	*128*	256	*128*	256	256	512	512	512	256	512
CMB	/	1024	512	512	>1024	512	>1024	>1024	1024	512	>1024
CMB/CMI	/	8	2	4	/	2	/	/	2	2	/
*D. cordata*											
CMI	>1024	>1024	512	512	>1024	512	>1024	>1024	>1024	>1024	>1024
CMB	/	/	>1024	>1024	/	>1024	/	/	/	/	
CMB/CMI	/	/	/	/	/	/	/	/	/	/	
*D. edulis*											
CMI	*256*	*256*	*128*	*64*	*64*	*256*	*256*	*256*	*128*	*128*	*256*
CMB	512	512	256	256	128	1024	1024	1024	256	256	1024
CMB/CMI	2	2	2	4	2	4	4	4	2	2	4
*E. speciosa*											
CMI	>1024	256	512	64	256	256	512	256	512	256	512
CMB	/	1024	1024	*128*	512	512	1024	>1024	>1024	1024	>1024
CMB/CMI	/	4	2	2	2	2	2	/	/	4	/
*K. crenata*											
CMI	256	512	256	256	256	512	>1024	>1024	*128*	*128*	256
CMB	512	>1024	512	1024	1024	1024	/	/	256	256	512
CMB	2	/	2	4	4	2	/	/	2	2	2
*O. gratissimum*											
CMI	256	*128*	256	*128*	256	256	256	512	512	*64*	128
CMB	512	256	1024	512	512	512	512	>1024	1024	128	256
CMB/CMI	2	2	4	4	2	2	2		2	2	2
*P. oleracea*											
CMI	>1024	512	512	>1024	>1024	>1024	>1024	>1024	>1024	512	>1024
CMB	/	1024	1024	/	/	/	/	/	/	1024	/
CMB/CMI	/	2	2	/	/	/	/	/	/	2	/
*S. dulcis*											
CMI	512	256	512	512	>1024	512	512	512	512	*128*	256
CMB	>1024	512	>1024	>1024		1024	>1024	>1024	1024	256	1024
CMB/CMI		2				2			2	2	4
*S. filicaulis*											
CMI	512	256	256	512	256	512	>1024	256	512	*128*	256
CMB	1024	1024	512	>1024	512	1024	/	1024	>1024	256	512
CMB/CMI	2	4	2	/	2	2	/	4		2	2
*S. veronicifolia*											
CMI	512	>1024	>1024	>1024	>1024	>1024	>1024	>1024	>1024	*128*	>1024
CMB	1024	/	/	/	/	/	/	/	/	512	/
CMB/CMI	2	/	/	/	/	/	/	/	/	4	/
Ciprofloxacine											
CMI	1,25	0.625	2.5	1.25	2.5	5	1.25	0.312	0.625	1.25	0.625
CMB	5	0.625	2.5	5	2.5	5	5	1.25	5	1.25	1.25
CMB/CMI	4	1	4	1	1	4	4	8	8	1	2

Staph: *Staphylococcus aureus, /* no activity

Considering all the inhibitory activity, MICs values of all the active plant extracts were almost fourfold less than their MBCs values.

## Discussion

The plants selected in this study are all used in Cameroonian traditional medicine to overcome a wide range of diseases. Ethno-pharmacological data have confirmed their role in health maintenance and promotion, but the major challenge is either to provide scientific evidence or to produce complementary data of their previous well established antibacterial properties.

Each of the extract of *D. edulis, O. gratissimum, S. dulcis*, *S. filicaulis,C. erecta* and *E. spciosa* tested in the present study displayed antibacterial activity on bacterial isolates tested. This evidence emphasizes the role of ethnopharmacological data as a framework for the discovery of bioactive compounds from plants.

Antimicrobial activity of plant extracts are routinely classified on the basis of susceptibility tests that produce MICs values in the range of 500–1500 μg/ml [24]. The activity is considered to be significant if MICs values are below 500 μg/ml and moderate when the MICs vary from 500 to 1500 μg/ml. Based on this scale, *D. edulis* was found to have significant antibacterial activity on all the 11 *S. aureus* isolates. *O. gratissimum* revealed similar activity on nine of the 11 isolates while *C. erecta* and *S. filicaulis* revealed similar results on 6 of the 11 clinical isolates. In general, MBC/MIC ratios less than or equal to four signifies a bactericidal effect of the test substance [15]. This indicates that the bactericidal effect of the active plant extracts could be expected.

Previous studies on the antibacterial activity of the essential oil of *Lippia sidoides* on clinical isolates of *S. aureus* had revealed a much important activity compared to other plants (MIC 400 µl/ml) [25]. The above plant extracts have proven much higher activities. Therefore these plants could be good candidates to overcome infectious diseases associated with *S. aureus*. These results are relevant since this microorganism is one of the most important human pathogens associated with hospital and community-acquired infections. Over the last few decades, the number and proportion of methicillin-resistant *S. aureus* infections in different countries has increased due to the rise of epidemics in humans [2, 26] and other animals, such as dogs, cats, cattle, pigs and exotic species [27].

*Dacryodes. edulis* and *O. gratissimum* activity are in accordance with previous work. Indeed, significant antibacterial activity of these plants on many bacterial species including *S. aureus* is well documented [10, 28–31]. Nevertheless, the real extend of this previous antibacterial results could not be compared to the present finding since the agar diffusion tests were performed.

The antibacterial activity of *S. dulcis* [32], *S. filicaulis* [33] and *K. crenata* [20] was earlier reported on Gram negative and Gram positive bacteria including *S. aureus*. Except *K. crenata* extract which revealed similar weak activity on *S. aureus* [15]. It was difficult to compare the limit as earlier mentioned. The present findings are therefore additional data that support the antibacterial activity of these plants as potent candidates to overcome infections associated with bacteria including *S. aureus*. To the best of our knowledge, the antibacterial activity of *C. erecta* and particularly on *S. aureus* is reported here for the first time.

The phytochemical screening was in accordance with reported data but slight differences were noted [15, 30, 34–36]. The phytochemical groups found in these extract could explain the antibacterial activity observed as well as the differences since the secondary metabolites of plants have many effects including antimicrobial properties [37]. Moreover, the differences could be attributed mainly to the chemical reaction method commonly used to identify the phytochemical groups of constituents. In fact, plant extracts are usually colored and this may mask specific color of some particular phytochemical group. The origin of the plant materials and the nature of the solvent for extraction are other factors that may affect the composition. Moreover, the distribution of these phytochemical groups varied from one organ to another.

## Conclusion

The present finding showed that *D. eduli, O. gratissimum,C. erecta* and *S. filicaulis* possess interesting inhibitory properties against *S. aureus* species. These data are promising and could encourage further researches on phytochemical, toxicological and pharmacological aspects of these extract-products in order to support their possible rational use in antimicrobial therapy, particularly, in anti-*S. aureus* therapy.

## Abbreviations

MIC: minimal inhibitory concentrations
MBC: minimal bactericidal concentrations
DMS: dimethylsulfoxide
INT: iodonitrotetrazolium chloride
